# Phenotype Heterogeneity in Glucokinase–Maturity-Onset Diabetes of the Young (GCK-MODY) Patients

**DOI:** 10.4274/jcrpe.4461

**Published:** 2017-09-01

**Authors:** Anna Wędrychowicz, Ewa Tobór, Magdalena Wilk, Ewa Ziółkowska-Ledwith, Anna Rams, Katarzyna Wzorek, Barbara Sabal, Małgorzata Stelmach, Jerzy B. Starzyk

**Affiliations:** 1 Polish-American Pediatric Institute, Jagiellonian University Collegium Medicum, Department of Pediatric and Adolescent Endocrinology, Cracow, Poland; 2 Polish-American Pediatric Institute, Jagiellonian University Collegium Medicum, Students’ Scientific Group at the Department of Pediatric and Adolescent Endocrinology, Cracow, Poland; 3 Equal main/first authors

**Keywords:** Glucokinase-Maturity-Onset Diabetes of the Young, GCK-MODY, children, adolescents, genotype, phenotype

## Abstract

**Objective:**

The aim of the study was to evaluate the clinical phenotypes of glucokinase-maturity-onset diabetes of the young (GCK-MODY) pediatric patients from Southwest Poland and to search for phenotype-genotype correlations.

**Methods:**

We conducted a retrospective analysis of data on 37 CGK-MODY patients consisting of 21 girls and 16 boys of ages 1.9-20.1 (mean 12.5±5.2) years, treated in our centre in the time period between 2002 and 2013.

**Results:**

GCK-MODY carriers were found in a frequency of 3% among 1043 diabetes mellitus (DM) patients and constituted the second most numerous group of DM patients, following type 1 DM, in our centre. The mean age of GCK-MODY diagnosis was 10.4±4.5 years. The findings leading to the diagnosis were impaired fasting glucose (IFG) (15/37), symptoms of hyperglycemia (4/37), and a GCK-MODY family history (18/37). Mean fasting blood glucose level was 6.67±1.64 mmol/L. In the sample, there were patients with normal values (4/37), those with DM (10/37), and IFG (23/37). In OGTT, 120 min glucose level was normal in 8, diabetic in 2, and characteristic for glucose intolerance in 27 of the 37 cases. Twelve of the 37 cases (32%) were identified as GCK-MODY carriers. In the total group, mean C-peptide level was 2.13±0.65 ng/mL and HbA1c was 6.26±0.45% (44.9±-18 mmol/mol). Thirty-two patients had a family history of DM. DM autoantibodies were detected in two patients. The most common mutations were p.Gly318Arg (11/37) and p.Val302Leu (8/37). There was no correlation between type of mutations and plasma glucose levels.

**Conclusion:**

The phenotype of GCK-MODY patients may vary from those characteristic for other DM types to an asymptomatic state with normal FG with no correlation with genotype.

**What is already known on this topic?**

Monogenic glucokinase-maturity-onset diabetes of the young (GCK-MODY) is the second most common type of diabetes mellitus (DM) after type 1 DM in a population of children and adolescents in Central Europe. Since it has been possible to genetically test patients with DM, the number of CGK-MODY patients in Poland has been increasing.

**What this study adds?**

This paper presents the detailed clinical presentation of GCK-MODY patients. Only 32% of all analyzed GCK-MODY carriers fulfilled DM diagnostic criteria, the rest presented with impaired fasting glucose or glucose intolerance. Our clinical data could help to identify GCK-MODY patients among patients with DM. The proper diagnosis could avoid insulin therapy in young patients which had previously been misdiagnosed as type 1 DM.

## INTRODUCTION

Maturity-onset diabetes of the young (MODY) is a monogenic form of diabetes inherited in an autosomal dominant way (1,2). There are over 800 known mutations associated with MODY and new ones are being discovered all the time (3). Glucokinase-maturity-onset diabetes of the young (GCK-MODY), also known as MODY type 2, is the most common type of the monogenic diabetes in Poland (4), and, along with a HNF1A- MODY, is one of the most common in the world. It is caused by a heterozygous mutation in the glucokinase gene on chromosome 7 (5). Glucokinase in the pancreatic beta cells senses increased blood glucose levels and controls the release of insulin. The heterozygous mutation in the glucokinase-coding gene results in a changed insulin threshold and therefore persistent hyperglycemia (6). As the hyperglycemia is mild and does not progress or cause any long-term complications, it may remain unnoticed (7).

GCK-MODY patients are usually non-obese, do not require treatment, and do not have vascular complications. This is the reason why it is important to differentiate this type from diabetes mellitus type 1 and type 2 (DM1 and DM2) in order to avoid unnecessary treatment (3,8,9,10). It should also be kept in mind that at the beginning, MODY was considered as a rare form of diabetes, however, it is probably much more common than assumed but often remains undiagnosed. By spreading knowledge of the existence of groups of diabetes such as MODY, and through the possibility of molecular testing, we should be able to change this situation.

The aim of this present study was to evaluate the clinical phenotype of GCK-MODY patients from Southwestern Poland treated in our department and also to search for phenotype-genotype correlations.

## METHODS

For this retrospective analysis, of all 1043 patients with DM treated in the Department of Pediatric and Adolescent Endocrinology in Cracow, we selected 37 (21 girls and 16 boys) aged between 1.92 and 20.1 years, with a mean age of 12.5±5.2 years, and with genetically confirmed GCK-MODY, which were included in the study. All participants and/or their parents gave their written informed consent to use their clinical data in scientific publications. All patients had been treated in our department in the years 2002-2013.

The following data were analyzed in details: age at GCK-MODY diagnosis, anthropometric data at diagnosis and during treatment, signs and symptoms at the time of diagnosis, medical history including course of pregnancy, birth parameters, and family history. Results of oral glucose tolerance test (OGTT), C-peptide, HbA1c, lipid profile, autoantibodies, and presence of other co-morbidities were also analyzed.

The clinical molecular testing was performed in an approved laboratory, with results interpreted by a board-certified clinical molecular geneticist or molecular genetic pathologist or the equivalent, in accordance with the American College of Medical Genetics (ACMG) Standards and Guidelines 2015 (11). The gene mutations were assessed in the Laboratory of Immunopathology and Genetics, Medical University of Lodz, Poland, which has achieved the International Quality Certificate ISO 9001:2008, a certificate of the Polish Society of Human Genetics. Molecular testing was performed by DNA sequencing performed using fluorescent-labeled terminating deoxynucleoside triphosphates with gene-specific oligonucleotide primers and multiplex ligation-dependent probe amplification to detect ezon deletions (MRC-Holland, Amsterdam, The Netherlands). Details of the molecular methods used in our patients were reported previously (4,12). The molecular testing was also performed in family members of patients including parents, siblings, and grandparents with impaired fasting glucose (IFG), glucose intolerance (GI), or DM.

Anthropometric measurements were taken in all patients. Height was measured to the nearest millimeter using a rigid stadiometer. Weight was measured to the nearest 0.1 kg using a calibrated balance scale. Reference data for Polish children were used for assessment (13). Body mass index (BMI) was calculated as weight in kilograms (kg) divided by the square of the height in meters (m^2^).

The statistical analysis was performed using Statistica/MS Exel programs. Student’s t-test was used to compare the analyzed groups. A p-value <0.05 was considered as statistically significant.

## RESULTS

GCK-MODY carriers amounted for 3% of all DM patients in our center (37/1043). The mean age at diagnosis was 10.4±4.5 years. The suspicion of GCK-MODY was based on different heterogeneous signs, symptoms, and results of laboratory tests. 14/37 patients presented with an IFG, 4/37 patients were admitted with symptoms of hyperglycemia (polydipsia, polyuria, fatigue), 1 patient was obese and presented with IFG, and 18/37 patients had had monogenic DM previously diagnosed in their family. Before the confirmation of GCK mutation, 9/37 patients were treated as DM1 and in 6/37 cases insulin had been administered.

The mean serum fasting glucose level was 6.67±1.64 mmol/L and the level ranged between 5.2 and 9.2 mmol/L. According to their blood glucose levels, the patients could be divided into three groups: those (4/34 patients) with normal values with a glucose level below 5.5 mmol/L (100 mg/dL), those (10/34 patients) with a glucose level above 6.9 mmol/L (125 mg/dL) characteristic for DM, and the remaining group (20/34) with an IFG 5.5-6.9 mmol/L (100-125 mg/dL). The profiles for OGTT results were also variable. The 120 min glucose level was normal in 8/26 patients and lower than 7.8 mmol/L (140 mg/dL). Two of the 26 patients had a result characteristic for DM, namely, higher than 11.1 mmol/L (200 mg/dL), and the remaining patients (16/26) had GI with values between 6.9 and 11.1 mmol/L (140-200 mg/dL). Thus, 12 of the 37 patients (32%) who were CGK-MODY carriers fulfilled the criteria of DM according to the Polish Diabetes Association recommendations (14). This figure is equivalent to 12/1043 (1.15%) of all patients with DM in our center.

The mean fasting C-peptide level was 2.13±0.65 ng/mL (normal ranges 0.2-4.2 ng/mL) and in 18/19 cases the result was above 0.75 ng/mL. Mean HbA1c level at diagnosis was 6.26±0.45 % (44.9 mmol/mol). This changed during the diet alone treatment into 6.13±0.39 % (43.5 mmol/mol) and the difference between the results was statistically significant (p<0.013). Six patients (6/37) had an elevated LDL cholesterol and five patients (5/37) had elevated total cholesterol levels. The mean levels of total cholesterol (4.58±1.02 mmol/L), LDL cholesterol (2.52±1.02 mmol/L), high-density lipoprotein (HDL) cholesterol (1.4±0.23 mmol/L), and triglycerides (0.99±0.35 mmol/L) were within normal ranges.

One patient was obese and one was underweight, the rest had BMI results normal for age. Two children (siblings) were diagnosed because of short stature and in both cases, pathological causes of short stature were excluded. Height of the other children were within normal ranges for age and compatible with their mid-parental height values.

Eighteen of the mothers of these children (18/37) had a confirmed GCK mutation, usually during family genetic tests. Two mothers (2/37) are being treated for DM2. Six mothers (6/37) were diagnosed with diabetes during pregnancy, found through routine screening, and all of them are GCK mutation carriers. In two of their children (2/37), birth weights were greater or equal to 4000 g. In both of these cases, children had the same GCK mutations as their mothers. Three patients (3/37) were small for gestational age babies (15). All these patients got their mutations from their fathers. Two of them were twins.

The patients presented highly incriminating family histories. In the case of 32 patients (32/37) one of the parents (18 mothers, 12 fathers) was diagnosed with diabetes GCK-MODY and had the same mutation as her/his child. A clear autosomal dominant mode of inheritance was presented. In five cases (5/37), the histories are not known. In an interview with 21 patients (21/37), diabetes also appears in one of the grandparents. The great-grandparents of 4 patients (4/37) had a confirmed GCK mutation.

Autoantibodies typical for DM1 such as islet cell autoantibodies (ICA), insulin autoantibodies, glutamic acid decarboxylase (GAD) autoantibodies, tyrosine kinase autoantibodies, were detected in 2 patients (2/37). One of these two patients had all four above-mentioned antibodies positive, while only ICA autoantibodies were positive in the second patient. Autoimmune diseases were not observed in this group of patients. Three patients (3/37) had Gilbert disease.

Thirteen mutations of the GCK gene were identified. Eleven (11) of these were missense mutations, one nonsense mutation, and one deletion (Table 1). The most common were p.Gly318Arg (11/36) and p.Val302Leu (8/36). Seven subjects with the second mutation (7/8) are members of the same family.

The variability of glucose levels was also observed within the carriers of the same mutation (p.Gly318Arg, p.Val302Leu).

Regarding the OGTT profile, we observed two different patterns in p.Gly318Arg mutation carriers. In 3 of these 11 patients, the mean increase in plasma glucose level was 5.56 mmol/L and the 120 min result was typical for DM, whereas in two other subjects (2/11), the mean increase was 1.45 mmol/L and at 120 min, the glucose level was within the normal range. At baseline, the glucose level was increased but similar in both groups (5.2-6.7 mmol/L) (Table 2).

A similar situation was observed in the carriers of p.Val302Leu mutation. Baseline glucose levels were elevated in all patients in this group, ranging from 5.5 mmol/L to 6.23 mmol/L (Table 3). Three patients (3/8) had a mean increase of 0.5 mmol/L in OGTT at 120 min and normal glucose levels, while two others (2/8) showed an increase of 2.95 mmol/L and the glucose levels suggest GI. Presented differences had no relationship with the age of the patients.

## DISCUSSION

GCK-MODY is mostly described as an asymptomatic condition, with mild fasting hyperglycemia (5.5-8 mmol/L), minor postprandial glucose extrusion, and a family history of diabetes. Usually, only a proper diet is sufficient to maintain an appropriate glucose level and prevent diabetic complications (1,8,9,10). However, new studies have revealed that GCK-MODY patients are not such a homogeneous group and that their phenotypes may vary considerably depending on the type of mutation (16,17,18). Results of these studies indicate that about half of GCK-MODY patients fulfill the criteria of DM, while the rest present with IFG or GI (16). Missense mutations have variable effects on glucokinase activity ranging from a small change in affinity for glucose to complete inactivity (19). Analysis of the clinical data of our patients led to similar conclusions. The mean features of the whole group were similar to those described in the literature, although findings pertaining to characteristics such as autoantibodies, obesity, fasting glucose levels were radically different in some of the patients from those characteristics for other types of diabetes. Compared to previous reports, we observed a lower percentage (32% vs. 50%) of GCK-MODY carriers fulfilling the criteria of DM. OGTT results of patients with two of the most frequent missense mutations (p.Gly318Arg, p.Val302Leu) show that their affinity for glucose does not correlate to the type of the mutation and even within the same mutation, postprandial glucose levels may vary significantly. There is no strong correlation between certain types of mutations and plasma glucose levels, although the threshold of insulin secretion is hypothetically the same. Differences in insulin sensitivity (20,21), diet, and physical activity might be probable reasons for those findings. Potential roles of other genes that modulate GCK function are also possible, considering that the GCKR regulatory protein gene has already been shown to interact with polymorphisms with GCK in a clinically significant way (22,23). GCK mutations affect not only the pancreatic function of this enzyme but the liver function too, where the decrease in the glycogen synthesis and storage, as well as increase in glucogenesis after standard meals, is reported. This defect in hepatic glucose metabolism contributes to postprandial hyperglycemia of GCK-MODY patients (24,25,26).

Our study reports the clinical presentation of 37 patients with confirmed GCK-MODY from a single, pediatric centre in Central Europe. The analysis of the clinical data includes a detailed process of diagnostics and also the assessment of the results of GCK-MODY treatment. The diagnosis of GCK-MODY was suspected on the basis of atypical signs and symptoms of diabetes, or atypical results of treatment, or previous family history of GCK-MODY. The limitation of the study is a lack of genetic tests in the whole group of 1043 patients with DM treated in our department due to financial limitations.

Recent studies report that specific gene mutations can present clinically as a neonatal form as well as ‘type 2‐like’ or ‘type 1‐like’ forms during adulthood (27) which makes diagnosis very difficult especially when symptoms, laboratory tests, and phenotype correspond to ‘double’ diabetes or even ‘triple’ diabetes with features of DM1, DM2, and presence of monogenic mutation. It is not an exception that patients with GCK-MODY are diagnosed as DM1 and treated with insulin. According to the literature, those subjects require higher than replacement doses to improve metabolic control of DM (28) and it may be a reason for their physicians to extend their diagnosis. In our group of patients, two boys presented with such inexplicably high requirement for insulin, whereas in another four children, the requirement for insulin was lower than that of other patients with DM1. These facts together with a strong positive family history of DM should also lead to a suspicion of monogenic diabetes.

Despite wide ranging glucose levels, HbA1c values were similar in all patients and mean levels were below the 10th percentile for diabetic children (22). Furthermore, we observed a significant reduction of HbA1c levels after treatment with diet alone. Reported fasting C-peptide levels above 0.75, persisting for 3-5 years from diagnosis, are suggestive of DM2 or monogenic diabetes. Similar values are consistent with short-term insulin independence in an individual who has not previously ‘failed’ non-insulin therapy but may occur in the DM1 diabetes honeymoon period (29).

The prevalence of dyslipidemia encountered in some patients may occur in GCK-MODY but is characteristic rather for DM2 (19,30). The carriers of GCK mutations usually show lower levels of fatty acids and triglycerides in circulation than the healthy population (31). Reduced GCK activity is likely to reduce glycolytic flux and production of both glycogen and malonyl-CoA. The latter is an important regulator of lipid metabolism; reduced levels alleviate inhibition of carnitine palmityl transferase 1, thereby increasing fatty acid oxidation. In addition, malonyl-CoA is the precursor of fatty acid synthesis; this will potentially also be reduced when GCK activity drops. Moreover, esterification of fatty acids into TAGs would be insufficient owing to reduced production of glycerol-3-phosphate via glycolysis. Thus, overall, hepatic fatty acid and TAG production and glucose metabolism would be decreased in the face of reduced GCK activity (31). Moreover, HDL cholesterol values measured in individuals with likely monogenic diabetes may be useful in screening for GCK-MODY and its differentiation from DM1 and HNF1A-MODY, regardless of treatment or metabolic control (12). The studies performed by Fendler et al (32) showed that individuals with GCK-MODY exhibit a strongly protective profile HDL cholesterol (high concentration of large HDL and low levels of intermediate and small HDL subpopulation). Next to constitutively moderately elevated glycemia observed in these patients, this lipid profile may be also a factor contributing to the low frequency of cardiovascular complications (32).

A twofold increase in incidence of Gilbert disease in our patients compared to the general population appears to be due to inter-family relationships in some of these individuals.

The positive GAD antibodies observed in two patients are not characteristic for GCK-MODY. The prevalence of GAD antibodies and a confirmed genetic diagnosis of MODY may represent the 1-2% of the population with detectable islet antibodies with no associated pathogenesis. In general, the finding of islet autoantibodies makes the diagnosis of MODY very unlikely, and genetic testing should only be performed if other clinical characteristics strongly suggest this form of diabetes rather than DM1 (33). Furthermore, positive diabetes autoantibodies can be transient in GCK-MODY patients and are not markers of prediabetes. It is possible that autoantibody titers are aggravated by obesity or by other factors, such as drugs (34).

Individuals with GCK-MODY are usually not obese and achieve normal growth. The imposition of obesity cannot be excluded, in view of the overall increase in obesity in all populations.

Large population cohort studies of pregnant women estimate the population prevalence of GCK-MODY as 1.1 in 1000 (35,36). The percentage of mutations in females diagnosed with diabetes in pregnancy could be significantly greater. Most importantly, maternal hyperglycemia in pregnancy is the primary risk factor for newborn macrosomia caused by fetal hiperinsulinism. GCK-MODY patients present with good, sometimes even high, insulin production and function but have increased set point stimulated insulin secretion. Usually babies with maternally inherited GCK-MODY and which have an increased set point stimulated insulin secretion, secrete a normal amount of insulin with maternal hyperglycemia, and have normal birth weights. Very high maternal hyperglycemia could result in fetal insulin hypersecretion, and ultimately, an overweight newborn, as was the case in two of our patients. The low birth weight observed in our three patients with paternally inherited GCK-MODY and non-diabetic mothers may be an effect of fetal hyperglycemia due to GCK mutation. Hyperglycemia in these cases is a result of low insulin secretion in the milieu of maternal normoglycemia due to increased set point stimulated insulin secretion.

According to the guidelines for GCK-MODY diagnosis, mild fasting hyperglycemia (5.5-8.0 mmol/L), small increase in OGTT (<4.6 mmol/L) at 120 min, negative autoantibodies, and MODY diagnosed in one of the parents are key indications for the genetic screening for GCK mutations. Our study shows that the diagnosis of this type of diabetes is more challenging in some cases. The phenotype of GCK-MODY patients may vary from one that is characteristic of other types of diabetes to an asymptomatic state with normal fasting glucose levels. Differences in insulin sensitivity, diet, and physical activity may be the probable causes of these findings. Potential roles of other genes modulating GCK function are also possible. The most important observation is that the proper diagnosis of GCK-MODY could lead to cessation of insulin treatment with improvement in the patients’ quality of life.

## Figures and Tables

**Table 1:**
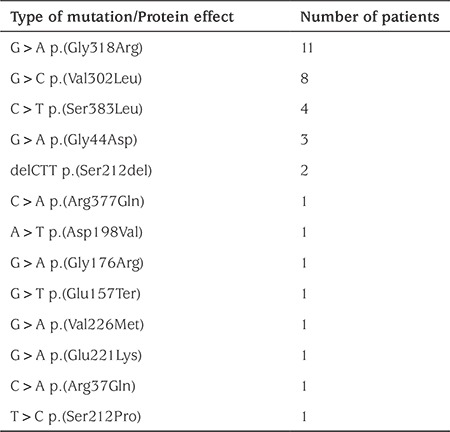
Types of glucokinase mutations in our patients

**Table 2:**
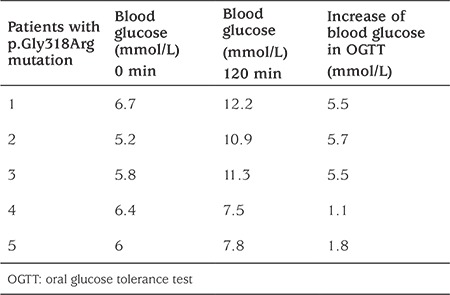
Oral glucose tolerance test profile in p.Gly318Arg mutation carriers

**Table 3:**
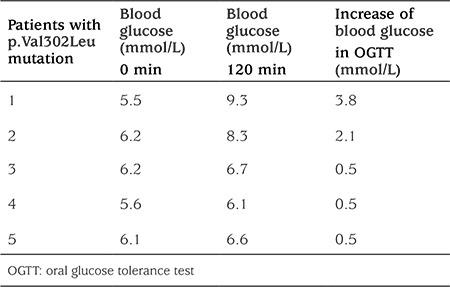
Oral glucose tolerance test profile in p.Val302Leu mutation carriers (blood glucose)
